# Donor information in research and drug evaluation with induced pluripotent stem cells (iPSCs)

**DOI:** 10.1186/s13287-020-01644-4

**Published:** 2020-03-19

**Authors:** Marcin Orzechowski, Maximilian Schochow, Michael Kühl, Florian Steger

**Affiliations:** 1 Institute of the History, Philosophy and Ethics of Medicine, Ulm University, Parkstraße 11, 89073 Ulm, Germany; 2 Institute of Biochemistry and Molecular Biology, Ulm University, Albert-Einstein-Allee 11, 89081 Ulm, Germany

**Keywords:** Induced pluripotent stem cells, Donor information, Information process, Ethics, Biobanks

## Abstract

**Background:**

The discovery of induced pluripotent stem cells (iPSCs) opened the possibilities for reprogramming cells back to a pluripotent state. Because of no apparent ethical issues connected with donation and derivation of biomaterial, iPSCs are considered as a research alternative to ethically highly disputed human embryonic stem cells (hESCs). However, the unique character of iPSCs leads to numerous ethical considerations, which mainly concern the issue of donor information and consent for the use of biospecimen in research and drug evaluation.

**Methods:**

For the purpose of this analysis, we conducted a review of the literature in the PubMed/MEDLINE and Web of Science databases. The search algorithm led to the identification of 1461 results. After removing duplicates and screening of title and abstract, 90 articles were found to be relevant to the study’s objective. Full texts of these articles were apprised and 62 articles were excluded at this step for not properly addressing the study’s objective. In the final step, 28 articles were included in the analysis. Analyzed were both research and non-research manuscripts published in peer-reviewed journals.

**Results:**

In the case of iPSC research, the information process should be guided by general frameworks established for research on human subjects but also by specific characteristics of iPSCs. We determined four main domains and 12 thematic subdomains that should be included in donor information. Our results show that majority of authors agree to the content of information with regard to the areas of general information, storage of cells, and protection of privacy. Two main issues that are discussed in the literature are donor’s consent for use in future studies and the process of donor information.

**Conclusions:**

Given the unique character of iPSCs and the possibility of their various uses in the future, the content of donor information should contain specific information central to iPSC research. Effective methods of communicating information to donors should combine written and oral information with the possible use of multimedia.

## Background

The discovery of induced pluripotent stem cells (iPSCs) in 2006 by Takahashi and Yamanaka opened possibilities for reprogramming virtually all types of somatic cells back to a pluripotent state [1]. The development of this technology has revolutionized stem cell research and paved new avenues for regenerative medicine [2]. First, iPSCs are applied for research as models of disease development, so called “disease-in-a-dish” [3]. Second, iPSCs provided new possibilities in drug screening for efficacy and potential toxicities [4]. Third, iPSC technology is widely applied in regenerative medicine for the development of stem cell-based therapies, including the therapies of the skin, heart, neural tissues, eye, and blood diseases [5].

In Japan, iPS-derived cells have already been safely used in retina transplant for one patient [6]; however, the study was suspended for the following patients due to the identification of mutations in the iPSCs [7]. In another clinical trial, iPSC-derived cardiomyocytes were grafted onto damaged myocardium in order to regenerate the heart’s muscle [8]. The outcome of this study shows positive initial results [9]. An ongoing study using allogeneic iPSC-derived mesenchymal stem cells reported a partial response in almost 90% of patients and a complete response in over half of cases. At present, no serious adverse events of safety concerns have been disclosed for this study [10].

The features as well as the research and clinical potential clearly show the unique character of iPSCs. They provide a ubiquitous source of pluripotent material that may be used in numerous areas, including the transplantation into humans or even the creation of human gametes. Many applications are still not a subject of contemporary research, and their potential is to be developed. This leads to a rapid growth in translational initiatives, research projects, and commercial biobanks [2].

Because of no apparent ethical issues connected with donation and derivation of biomaterial, iPSCs are considered as a research alternative to ethically highly disputed human embryonic stem cells (hESCs) [11]. However, despite this view, the unique character of iPSCs leads to numerous ethical considerations. These challenges mainly concern the issue of donor information and consent for the use of biospecimen in research.

Robust consent procedures increase subjects’ autonomy and reduce their insecurities regarding the transparency and the actual goal of the research and its methods. They could also build up donors’ trust to researchers and can establish a relationship beneficial for both groups. Therefore, the content and process of donor information plays a central role in iPSC research. Consequently, our research focused on an examination of the existing body of literature on the topic of donor information in research and drug evaluation in order to provide an answer to the following questions: What is the specific information that should be included in the donor information process during the acquisition of biological material for research and drug evaluation with iPSCs? How should the process of donor information in this area be conducted?

## Methods and materials

### Methods

For the purpose of this analysis, we conducted a review of the literature in the PubMed/MEDLINE and Web of Science databases. The goal of this search was to provide answers and empirical evidence for the proceeding research questions. To ensure rigor in reporting, this study protocol followed the relevant aspects described in the Preferred Reporting Items for Systematic Reviews and Meta-Analysis (PRISMA) statement [12]. The examination was conducted using the following steps: (1) identifying a research question, (2) identifying relevant studies, (3) study selection, (4) charting the data, and (5) collating, summarizing, and reporting the results.

The search algorithm combined keywords “induced pluripotent stem cells (iPS cells)” or “human-induced pluripotent stem cells (hiPSCs)” with keywords “information process”, “patient information”, “informed consent”, and “ethics” in the title and abstract of the articles in databases. We selected papers published in the years 2007–2019, that is, since the publication of the first research results on the possibility of utilization of human-induced pluripotent stem cells.

This search algorithm led to the identification of 1461 results. After removing duplicates and screening of title and abstract, 90 articles were found to be relevant to the study’s objective. Full texts of these articles were apprised and 62 articles were excluded at this step for not properly addressing the study’s objective. In the final step, 28 articles were included in the analysis. The content of the articles was analyzed in order to extract the most important information pertaining to the study’s research question.

### Materials

Both research and non-research manuscripts from peer-reviewed journals were included in the analysis. Research manuscripts comprise research studies. Commentaries and case reports were the acceptable non-research manuscripts. The material for the analysis encompassed 11 commentaries and guidelines, 12 reviews, and five papers with study results.

## Results

Based on the analysis, we have identified four main domains and 12 thematic sub-domains that should be included in donors’ information in iPSC research and drug evaluation. An insight into these areas is provided in Table 1. These four main domains determine the following structure of this section.

**Table 1:** Overview of the main domains and sub-domains of the patient information in research on iPSCs

Domains	Topic of information
1. **General information for the donor**	
Study’s background information	The aim and method of the study
	Collection of donor’s personal data
	Biospecimen’s collection
	Contact information
Genetic modification	Genetic modification of the biomaterial in the course of the study
Commercial potential of the research	Property of body tissues
	Financial compensation for the donor
	Commercial result of the research
	Individual and commercial interests of the researchers
2. **Storage of the cell lines and protection of privacy and confidentiality**	
Storage of cells and cell lines	Information on the possibility of indefinite storage
	Destruction of the biomaterial
	Storage in repositories
Protection of privacy and confidentiality	Association of biomaterial with particular individuals
	Possibility of reidentification
	Data sharing
	Measures for the protection of donor’s privacy
Incidental findings	Possibility of acquisition of incidental medical findings
	Procedures for the return of incidental findings
3. **Research with the use of biospecimen**	
Future research	Use of biomaterial in other studies
	Acquisition of the consent for other studies
Reproductive research	Use of biomaterial for creation of gametes and embryos
	Use of biomaterial for the creation of human clones
Transplantation of cells and organs	Use of biomaterial in regenerative medicine
	Use of biomaterial for growing human organs
Research on animals	Grafting of iPSCs into non-human animals
	Creation of human-animal chimeras
4. **Process of donor information**	
Provision of information	Methods of donor information
Clarification of information	Evaluation of donor’s understanding of provided information

### General information for the donor

General information should include the purpose, method, and duration of the study [13]. Future medical and societal benefits, both for the participants and for other groups, can be outlined as well as potential uses of iPSCs [14]. The extent of personal and medical data that will be collected for the research, e.g., age, sex, and family history of the disease, ought to be specifically provided [15]. Donors should be aware of their rights and circumstances for the withdrawal from the study [13]. Information on the kind of donation required, e.g., skin cells or hair sample, the methods applied to derive the body cells, and the number and frequency of visits during the course of research should be included [15, 16]. Moreover, information about whether the procurement of biomaterial involves harm, pain, or social risks for the donors and about treatment provided in case of damage should be incorporated [17]. Such redress could involve financial compensation provided by the research’s insurance or the possibility of medical treatment financed by the researchers’ insurance coverage. Contact possibilities for donors’ inquiries should be included [15]. Moreover, some information on the unique nature of the human genome should be a part of the general information for the donor [14].

Several authors pay attention to the possibility that donor’s iPSCs and cell lines will be genetically modified in the course of research [18, 19]. The use of retroviral vectors in the reprogramming of iPSCs carries a risk of cancerous transformation. Therefore, in order to avoid the risk of viruses, the majority of iPSC laboratories currently use episomal vectors to generate clinical-grade iPSCs. However, information about such risk may be important for the donor as, in case of planned autologous interventions, it implicates individual risks. Such information allows donors to make a conscious decision about individual health and participation in the study. Once differentiated cell derivatives of iPSCs are transferred into a body, they cannot be metabolized or excreted and the negative consequences may not become apparent for decades [20].

One of the important aspects of iPSC research concentrates on its commercial applications. Connected to this area are the aspects of patenting scientific discoveries, developing commercial therapies, and of distribution of profits from the future commercialization of the research. The central question here is to whom the property of body tissues or genetic matter belongs after the moment of donation [20]. Patient information should include whether there will be direct financial compensation to the donor for participation in the research and a clause about payment in case of future products derived from the donation [15, 19]. Donors should be informed about the commercial interests of the researchers conducting the study, institutions, sponsoring companies, and referring physicians [14].

### Storage of the cell lines and protection of privacy and confidentiality

iPSCs and cell lines are immortal and can be stored indefinitely. With regard to storage and banking of biospecimens, donors should be informed about the possibility that their samples, iPSC lines, and relevant data will be placed in a repository (biobank) and about the purpose of the repository [14]. The consent process should include information concerning repository’s governance and review policies, as well as the timeline for banking and for using a contributed specimen. An explanation that the repositories holding the cells can distribute them to other institutions should be provided to donors [21].

iPSC lines, similarly as donor’s cells and tissues, carry a “genetic fingerprint” with an immeasurable amount of information [22]. Large-scale genome sequencing could lead to association of the medical data with a specific individual not only on the basis of donated cells but also on the basis of modified cell lines. As a result, it could lead to discrimination, stigmatization, stress, or anxiety [23, 24]. Therefore, information about the possibility of access to specific personal data, beyond the data directly provided by the donor, should be included. Additionally, on the basis of the genotype carried in iPSC lines, donors can be reidentified through matching genomic data against a reference in genotype or by profiling genomic data from DNA analysis [23, 25]. Donors should be informed about these risks. In order to preserve transparency, donors should receive information about standards and measures of data protection [14]. A description of risks towards the protection of confidentiality and measures that will be taken to minimize these risks should be required elements of informed consent. In addition, researchers should avoid giving tissue donors a guarantee of absolute anonymity or privacy [26].

In case of future use of biomaterial in a wide range of research, the possibility exists that donor’ relevant medical information will be obtained, e.g., predisposition to the disease. For the return of such incidental findings, their management should already be specifically addressed in the information process [27, 28].

### Research with the use of iPSCs

iPSCs can be used worldwide in various studies encompassing different methods and research aims, most of which may not have even been conceived in the moment of donation [19]. In case that acquired cells or tissues will not be used for the purpose of only one study and destroyed afterwards, the donor should be explicitly informed about this fact. Some of the reviewed authors propose that donors should be informed about specific areas of future research [29, 30]. This may have an influence on donors’ participation in the research. Although donors could generally agree to research based on their cells and cell lines, they might object certain research projects and refuse to donate cells for these projects [18].

In case of research with reproductive purposes, several authors agree that information about such a possibility should be the object of the information process. The aspect of reproductive research could raise serious ethical objections in donors, especially regarding the issues of creation and destruction of embryos during the research or with regard to the creation of human clones [18, 22, 29]. Similarly, donor information should contain the possibility of using iPSCs in therapeutic or regenerative medicine [14]. The idea that one’s cells or organs with one’s genetic origin are an integral part of another person’s body could require deeper reflection. It could even be outright rejected on grounds that transferred cells and organs will form an integral part of the recipient’s genome and can be passed on to future generations.

Furthermore, donors should be informed whether iPSCs will be used in research in which they will be grafted into non-human animals or whether they will be used for the creation of human animal-chimeras [17, 18, 31]. Injecting iPSCs into animals could provide valuable insights into the development and therapy of several conditions, for example, for preclinical testing of therapies for Parkinson’s disease or Alzheimer’s disease [25]. Yet, donors may oppose research on animals entirely or donors’ religious convictions may stand against mixing human and animal species [22].

### Process of donor information

The majority of the authors concentrate on specific information that should be provided in the process of obtaining informed consent. Little attention is paid in the reviewed literature on how the process should look like in the case of iPSCs. Standardized methods with the use of information sheets and brochures achieve poor understanding among participants of iPSC research [14, 32, 33]. Therefore, authors plead for alternative approaches including multimedia. Other possible recommendations include the use of videotapes, photographs, or diagrams of research procedures, pre-visits to the research site, group discussions, Web sites, and comics that explain the nature of the research [15]. Yet, such visits may involve health and safety concerns or involve additional costs and complex organization, especially in cases when the research site is geographically distant from the specimen collection points.

In addition, professionally trained staff should be responsible for the provision of information, in order to deliver information in a way that is tailored to the individual needs and perceptions of the donors [27]. Another suggestion is the involvement of stakeholder groups in designing the process of informed consent and in proposals for the amount of information that should be included in it [21].

An important point of the information process is a clarification of information that could be misunderstood or incomprehensible. Therefore, the authors argue that donors should be explicitly invited to ask questions [18]. The level of information comprehension should be assessed after the process of patient’s information in order to evaluate the actual understanding of the provided information [32, 34].

## Discussion

Studies on donors’ attitudes towards iPSC research show that they in general have a positive perspective towards this area of research [29]. However, considering the specific nature of iPSCs, ethical concerns might arise with regard to informed consent. In case of iPSC research, the information process should be guided by general frameworks established for research on human subjects but also by specific characteristics of the iPSCs. A sound and well-prepared consent process could contribute to safeguarding violations of personal autonomy and to avoidance of ethical pitfalls. It enables donors to make their own risk-benefit assessment and, on this basis, to make an autonomous decision about participation in the research [35]. Proper information in case of iPSC research can contribute to a successful research process that benefits both donors and scientists. Donors might benefit from research in several ways. First, based on altruistic reasons, they individually consider the purpose of the research as important or recognize their participation as fulfilling a social obligation in progressing medical knowledge. Second, because of research-related reasons, they might be interested in the research topic. Third, egoistic reasons would involve personal motivation, i.e., the possibility of finding a cure for an illness they suffer from or additional motivation through financial incentives. Benefits for researchers could result from multiple factors—from research-specific, such as interest in the topic and possible sponsorship to individual factors, such as advancement of medical knowledge and their own careers.

Our results show that the majority of reviewed authors agree to the content of donor information with regard to the areas of general information, storage of cells, and protection of privacy (domains 1 and 2). These results coincide with conclusions of studies on informed consent in research using genetic material [36]. Two main issues that are discussed in the debate on donor information in iPSC research are the issue of donor’s consent for the use of their biomaterial in future studies and the process of donor information (domains 3 and 4).

With regard to the issue of informed consent for future studies, the balancing of patients’ right to an autonomous decision and sustaining the process of scientific progress becomes central. Approaches to the issue of informed consent may vary from ongoing interaction with participants to a one-time agreement for the broad use of biomaterial [37]. In this context, balancing of donor’s right to an autonomous decision and ensuring scientific progress is a challenging task. On the one hand, informed consent requires specific permission for studies using donor’s material. Researchers should respect donor’s autonomy and the right to participate in research. The expression of individual autonomy is not static; it involves decisions that could change over a person’s lifespan [38]. Blanket or general consent to unspecified future studies could violate the main components of informed consent. Therefore, donors should be enabled to prospectively control the use of their specimen [39]. On the other hand, scientific research contributes to the benefit of society and patient. In the case of iPSC, it is difficult to obtain informed consent for all future research. Unnecessary delays or administrative burdens could decrease the effectiveness of the research. Obtaining new individual consent for each new use of the sample would lead to delays or even to an impediment of the research [40, 41]. Curbing the scientific progress due to undue delays and overwhelming administrative burdens will compromise the advancement of science. Thus, donors should provide their agreement to a broad variety of research. Other positions postulate the distribution of derived iPSCs, even after a donor withdraws from the study, however only in deidentified form [14].

In our opinion, the strategy of broad agreement in a single interaction with the donor could violate the donor’s autonomy, as there are reasons to assume that donors would not clearly realize what such consent entails. Therefore, the possibility to recontact the donor should have a central place in the information process. It would facilitate sharing of individual or aggregate research findings and incidental findings as well as to receive updated information about the health status of the donor [28]. In case of particularly compelling scientific reason and when it is impossible to contact the donor, an anonymized distribution of the biospecimen could occur with the agreement of the appropriate research oversight body [19].

A possible suggestion would be a tiered consent process. It involves asking donors during the initial consent to agree with further, not yet specified projects, i.e., reproductive research or transplantation into humans [18]. Such an approach, however, still follows a static expression of autonomy. Therefore, Kaye et al. suggest a dynamic consent approach. Dynamic consent is a personalized, secure, digital communication interface that allows researchers to engage donors through information technology (IT) means [38]. Using such interface, donors can tailor and manage their own consent preferences. They can give and revoke consent to the use of their samples in response to changing circumstances. It also allows a two-way communication between researchers and donors. Donors can be approached for consent to new projects or informed if new ethical questions arise in already ongoing projects. Dynamic consent can contribute to the improvement of transparency and public trust in projects, benefit recruitment, and enable more efficient participant recontact [42].

Ethical principles for medical research with human subjects clearly state that every research subject has the right to withdraw consent to participate at any time without reprisal, regardless of whether the research is conducted on the patient itself or on the biospecimen provided by the patient [43]. Consequently, there should be a realistic opportunity for the withdrawal of consent for those who have donated identifiable samples and consent for original research and who are not willing to re-consent in other subsequent research. However, while this right needs to be safeguarded, there is also a need to guarantee the quality of study results and prevent wasting of time and resources [44]. Therefore, during the information process or when asked to re-consent, donors should receive information about the critical importance of the provided biospecimen for the study’s outcome.

Provision of financial compensation for the donor might raise ethical concerns, especially with respect to the potentially coercive character of incentivizing [45, 46]. Incentives that encompass payments or rewards can, on the one hand, encourage donors to participate in research. On the other hand, they can lead to undue inducements if an incentive is so attractive that it impairs subject’s ability to exercise proper judgment [47]. In case of iPSC research, small incentives for the donors can have a positive effect on participation. However, a promise of payments from future products may encourage individuals to enroll in clinical trials for wrong reasons. Therefore, such practice should be avoided in order to prevent a donate-for-profit culture. It is important that donor information in this respect is transparent and describe any system of payment or reimbursement in detail.

The process of donor information plays an important role in encouraging participation in the study. Clearly stated research’s objectives and methods presented by trained recruiters could positively influence donors’ participation in the study [48]. Moreover, the use of educational videos or interactive computer presentation of trial information together with written information could increase donors’ knowledge about the study and their willingness to take part in it. In addition, real-time contact with researchers using social media might have a positive impact on participation [49, 50]. The key benefits of the use of social media involve increasing the number of interactions with researchers and the provision of personalized information tailored to donors’ needs. Social media can also provide communication in real time at relatively low cost, which can have a considerable influence on the donors’ comprehension of information. Disadvantages of the use of social media involve potential information overload, possible security breaches that could lead to a disclosure of donors’ personal information, and, in some cases, lack of privacy [51]. Moreover, the use of social media can be influenced by factors such as age, personality, literacy level, and cognitive abilities [52]. Therefore, although social media can improve the process of donor’s information, they should rather play a complementary and not the main role in the process.

Regarding the issue of the information process, the topic of adequate and effective communication is central. Reviewed authors agree that in order to facilitate the donors’ informed decision-making, risks and benefits should be clearly communicated. Donors should understand the specificity of iPSC research and implications of the donation for them as well as for the research and society. However, in which actual way donors of iPSCs should be informed and voluntary consent should be acquired remains unclear in the reviewed literature. Inadequate understanding of informed consent has been observed both in research [36, 53] and other areas of medicine [54]. Given the level of complication of the iPSC research, its methodology, and implications, standard procedures of donor information may not achieve the assumed aim. Moreover, it has been observed that the donor’s perceived understanding of provided information varies from actual understanding [55].

Therefore, in our opinion, alternative methods of information that contribute to a better understanding of information in iPSC research should be adopted. These could incorporate the use of multimedia (i.e., information videos or computerized presentations) and interactive tools (i.e., examination of information provided during the information process in the form of a questionnaire with subsequent feedback) [56]. This could allow to estimate the level of actual understanding. In addition, extended person-to-person contacts, i.e., through direct personal communication with a qualified person, discussion in groups, and the use of social media applications could provide improvement of the process. As the production of multimedia resources is costly and time-consuming and may not provide expected results if used alone [57], a combination of these three methods could provide the best outcome. In contrast, the provision of additional information in the form of an extended information sheet is unlikely to have a large effect on the quality of information process as it only involves the reading of the form without subsequent discussion of the provided information. In this context, some voices call for the decrease in the amount of information provided [58]. Otherwise, the process could become too burdensome and too complex. As a result, it could decrease the level of participation [21].

Application of intellectual property rights in the area of iPSC research and development can present a major challenge in the future [13]. Patent law should seek a balance between rewarding and promoting innovation and preserving the freedom of research. The proliferation of patents may potentially frustrate further research or translation of the technology. In case of iPSC research, important issues include questions whether derivation methods of iPSCs differ from the derivation processes of hESCs, which are at the moment ineligible for patenting in several countries [59]. Another key issue is whether various methods of reprogramming of iPSCs are patentable or whether they constitute an uninventive modification of already patented processes [22]. With regard to the donors, the question arises whether they should be entitled to share fruits of the successful research not only in the form of access to new forms of treatment or acknowledgement. Lack of financial recognition in the patent regime could lead to a sense of exploitation and decreased participation in research [60]. Therefore, the issue of just distribution of patent rewards between research institutions, scientists, and donors requires extended ethical, legal, and social discussion involving all stakeholders.

A challenge for the use of iPSC in the context of biobanking remains the issue of immunosuppression as donor cells express their own human leukocyte antigen (HLA) proteins. The creation of allogeneic iPSC banks could significantly reduce the cost for iPSC-based therapies [61], as only a relatively small number of samples would be required to match a large portion of the population [13]. For genetically homogenous countries, the creation of an allogeneic cell bank could provide a viable solution to this question [62, 63]. In case of more genetically heterogenic countries, a similar bank may be cost-prohibitive [61].

## Conclusions

Given the unique character of iPSCs and the possibility of their various use in the future, ethical issues connected to donors’ informed consent gain the central position in this area of medical research. Proper information content should contain specific information fundamental to iPSCs research. Donors should receive detailed information about the commercial potential of the research, storage of their biomaterial, and about protection mechanisms of personal data. Limitation of donor’s autonomy through broad consent procedures should be avoided. In order to improve comprehension of the information material, innovative methods of information, with the use of multimedia instruments, could prove to be helpful.

## Data Availability

Not applicable

## Abbreviations

hESC: Human embryonic stem cell
hiPSC: Human-induced pluripotent stem cell
HLA: Human leukocyte antigen
iPSC: Induced pluripotent stem cell
IT: Information technology
